# Application of the Migraine Aura Complexity Score (MACS): Clinical and Neuroimaging Study

**DOI:** 10.3389/fneur.2019.01112

**Published:** 2019-10-18

**Authors:** Igor Petrusic, Michele Viana, Marko Dakovic, Jasna Zidverc-Trajkovic

**Affiliations:** ^1^Laboratory for Advanced Analysis of Neuroimages, Faculty of Physical Chemistry, University of Belgrade, Belgrade, Serbia; ^2^Headache Center, Institute of the Neurocenter of Southern Switzerland (NSI), Regional Hospital Lugano, Lugano, Switzerland; ^3^Headache Group, Department of Basic and Clinical Neurosciences, King's College London, London, United Kingdom; ^4^Faculty of Medicine, University of Belgrade, Belgrade, Serbia; ^5^Center for Headaches, Neurology Clinic, Clinical Center of Serbia, Belgrade, Serbia

**Keywords:** migraine with aura, higher cortical dysfunction, dysphasia, cortical thickness, magnetic resonance imaging

## Abstract

**Background:** Manifestations of typical migraine aura can be numerous. Investigation of its pathophysiological mechanisms can be challenging if a stratification of phenotypes is not performed. In this context, the Migraine Aura Complexity Score (MACS), recently developed, may help. Here we aimed to categorize migraine patients into homogenous groups using MACS and to compare those groups with respect to patients' characteristics and neuroimaging findings.

**Methods:** Participants who have a migraine with aura (MwA) were interviewed after each attack in order to obtain the characteristics of migraine aura. Thereafter, we scored the complexity of their auras by MACS. The MACS was used to categorize patients into three groups: MwA-S (with simple aura), MwA-MC (with moderately complex aura), and MwA-C (with complex aura). The patient characteristics and estimated cortical thickness of regions of interest, which are potentially linked to the symptoms that develop during the aura, were used to compare these groups.

**Results:** In total, 338 MwA attacks were recorded in analyzed groups. Scotoma was the most frequently reported symptom in the groups, followed by somatosensory aura in the MwA-C group and zig-zag lines in the MwA-MC and MwA-S groups. Patients in the MwA-C and MwA-MC groups had a thicker cortex in the left primary visual cortex with respect to MwA-S group. In addition, patients in the MwA-C group had a thicker cortex in several visual and somatosensory cortical regions relative to the MwA-S group.

**Conclusions:** Our results show that the newly developed MACS can be used for the stratification of MwA patients, herewith allowing the better investigation of changes in migraineurs' brains.

## Introduction

Migraine with aura (MwA) strikes nearly 3.6% of the world population (1–3). In typical migraine aura (MA), visual symptoms are the most common, followed by somatosensory, and then dysphasic auras (4). Manifestations of visual auras can be numerous, including positive and negative symptoms, as well as disturbances of visual perception (5). Somatosensory symptoms can be manifested as tingling or numbness, which can lead to dyspraxia. Different forms of dysphasia and other higher cortical dysfunctions (HCDs), such as disturbances of memory, were noted during the MA (6). MwA becomes particularly important when the duration of neurological symptoms and the modality of their appearance may constitute a cause of severe anxiety and distress in patients (7).

MA is thought to be caused by cortical spreading depolarization followed by cortical spreading depression (CSD) (8). It is possible that propagation of CSD results in a variety of symptoms correspond to the affected cortical region (9). The widely accepted approaches to explore mechanisms of MA are different modalities of advanced neuroimaging (10–12). However, the main methodological issue of the majority of the studies lies in the lack of inappropriate homogenization of patients with respect to aura phenotype (13).

Recently, we developed a scoring system for evaluating the complexity of MA (Migraine Aura Complexity Score—MACS) with an aim to provide better stratification of MA patients who participate in neuroimaging studies or clinical trials (14). The range of MACS is 0–9. Higher values indicate more complex aura. We demonstrated that this score positively correlates with cortical thickness of some regions, which are potentially linked, from a functional point of view, to the symptoms that develop during the aura. Moreover, we found that MACS allows detecting patients who have a complex aura with a sensitivity of 86% and specificity of 100% if their median MACS after 10 recordings is ≥4.5 points. However, some of the issues relatives to the use of this scoring system have remained to be elucidated. In fact, categorization of patients whose median MACS is <4.5 points due to the fact that the majority of their auras are simple, but they also have few attacks with the score that denote them as patients with complex aura, is not determined. We hypothesize that these patients could have a different phenotype from those who experience mostly complex auras or those who have never experienced a complex aura. Furthermore, categorizing patients using MACS can be used to investigate patient characteristics in order to determine their phenotypes.

The aim of this study was to use MACS and three distinctive manifestations of typical aura (visual, somatosensory and dysphasic symptoms) to categorize migraine patients into homogenous groups and to compare them in terms of patients' characteristics, thus to investigate the clinical phenotype of patients who are stratified in the same group. Also, we aimed to explore the application of MACS in magnetic resonance imaging (MRI) studies that investigate the thickness of the cerebral cortex.

## Materials and Methods

### Participants

Migraine patients included in the study were from the cohort of patients that were enrolled in previous migraine neuroimaging studies (13, 15), including patients that participated in developing the MACS (14).

All participants had an episodic migraine with aura according to the International Classification of Headache Disorders criteria (3rd edition) (4). The inclusion criteria were: (a) individuals with episodic migraine, (b) 21–60 years of age, (c) acceptance of participation in the study, (d) absence of migraine preventive therapy, and (e) no pathological findings on participants' MRI scans. Exclusion criteria included a presence of other neurological or cardiovascular diseases and motor aura symptoms.

**Figure 1:**
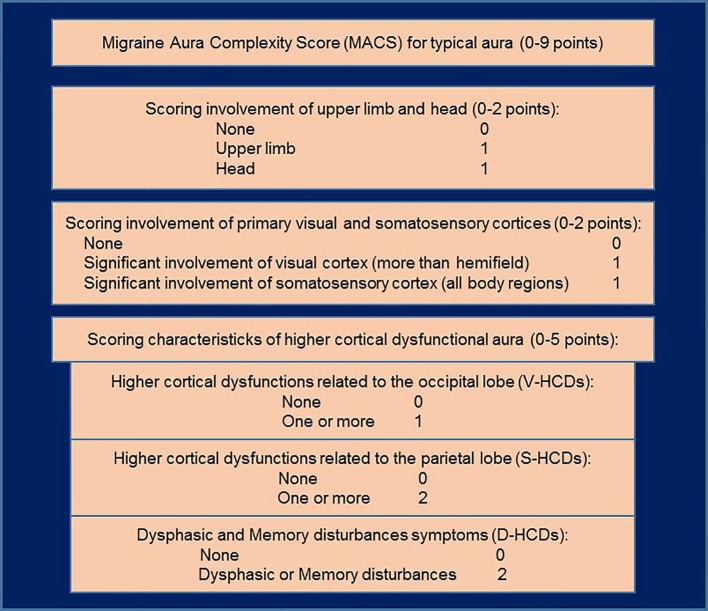
Schema of the Migraine Aura Complexity Score (MACS). Higher cortical dysfunctions of occipital cortex (V-HCDs): micropsia, macropsia, dysmorphia, fractured vision, and prosopagnosia; higher cortical dysfunctions of parietal cortex (S-HCDs): astereognosis, dyspraxia, and unawareness of one's own body parts; dysphasic and memory disturbances symptoms (D-HCDs): (Broca's dysphasia, Wernicke's dysphasia, dysnomia, dyslexia, difficulties in remembering or recalling events, recalling names, and calculating and/or memorizing numbers). Adapted from our previous research paper (14).

Selected participants were instructed to complete the specific questionnaire about the quality of aura symptoms after each attack of a MA (Table 1). Patients who had experienced visual disturbances also reported the level of involvement of the visual field (a quarter, half or the whole of the visual field), while patients who had experienced somatosensory symptoms also reported the number of body regions that were involved. Body regions were divided into three areas: (a) upper limb, (b) head, and (c) trunk/lower limb. Also, patients reported the duration of the aura and their subforms, duration of the headache, and pain intensity. The questionnaire was used to score the MACS (Figure 1) and to collect the characteristics of MA and headache. The questionnaire was filled out within 2 days after the attack to minimize a possible bias of failing to recall symptoms during attacks. The patients were monitored and data were collected during a period of 12 months (December 23rd 2017–December 24th 2018). In order to complete the study patients needed to record at least 6 MwA attacks during the monitored period allowing us to more accurately assess the overall MACS in each patient.

**Table 1:** Study questionnaire.

**During the aura of your migraine attack, have you noticed:**	
1.	Flashes of bright light in the visual field?
2.	Blurred spot in the visual field?
3.	Scotoma (a partial loss of vision)?
4.	Twinkling zig-zag lines in the visual field?
5.	Tunnel vision (narrowing of the visual field)?
6.	Deformed or deformed images, unrelated to the disturbance of vision?
7.	Difficulties in recognizing faces, unrelated to the disturbance of vision?
8.	Objects becomes biger or smaller?
9.	Tingling or numbness in hand, leg, and face (head)?
10.	Difficulties in recognizing objects by touch?
11.	Difficulties in activities requiring coordination and movement of extremities?
12.	Unawareness of one part of your body?
13.	Difficulties in recalling names?
14.	Difficulties in recalling or remembering events from the past?
15.	Difficulties in speaking even when you knew what you wanted to say?
16.	Difficulties in understanding people who were talking to you?
17.	Difficulties in reading comprehension, unrelated to visual disorders?
18.	Difficulties in writing that were not caused by the disturbance of vision?
19.	Difficulties in calculating and/or memorizing numbers?
If you expirienced symptoms of visual aura please report the level of involvement of the visual field (a quarter, half or the whole of the visual field):	
How did your visual aura symptoms last for?	
If you expirienced symptoms of somatosensory aura please report the number of body regions that were involved (upper limb, head and/or trunk/lower limb):	
How did your somatosensory aura symptoms last for?	
If you expirienced symptoms of dysphasic aura please report the duration:	
How long was the duration of a headache?	
Please rate head pain intensity on the scale from 1 to 10:	

The MACS was used to categorize patients into three categories: (1) patients who have simple auras (MACS ≤ 1 point), (2) patients who have moderately complex auras (MACS between >1 and <4.5), and (3) patients who have complex auras (MACS ≥4.5 points), making MwA-S, MwA-MC, and MwA-C groups, respectively. Also, patients were stratified into visual (patients who have only visual symptoms), somatosensory (patients who have visual and somatosensory symptoms) and dysphasic (patients who have visual, somatosensory and dysphasic symptoms) groups, making MwA-V, MwA,-SS, and MwA-D groups, respectively. The patient characteristics and neuroimaging measures of cortical thickness were used to compare these groups.

### MRI Data Acquisition

MR examinations of patients were performed using a 1.5 T MR scanner with an eight-channel head coil (Signa, General Electric Healthcare, Milwaukee, WI, USA). The imaging protocol consisted of T2 weighted spin echo (T2W) in an axial plane [Echo time (TE) = 105.8 ms, repetition time (TR) = 5,700 ms, flip angle (FA) = 90°, 24 slices with 0.47 × 1 × 5 mm^3^ voxels, slice thickness = 5 mm, acquisition matrix 512 × 512) and three-dimensional T1 weighted fast spoiled gradient-echo (T1-3D-FSPGR) series (TE = 3.60 ms, TR = 8.12 ms, FA = 15°, 248 contiguous slices with 0.47 × 0.47 × 1.4 mm^3^ voxels, slice thickness = 1.4 mm, acquisition matrix 512 × 512, FOV = 256 × 256 mm^2^). T2W images were only used to exclude the presence of brain lesions.

Freesurfer (version 5.3.0) analysis was performed on an HP 350 server (Intel Xeon 1,800 Mhz, eight cores, 16 GB RAM) using a recon-all script for automatic cortical reconstruction and segmentation of brain structures. Average run time (with the parallelization option used) was 6 h. Details about Freesurfer and its routines can be found elsewhere (16, 17). For this study, we used cortical thickness measures of predefined cortical regions of interest (Primary visual cortex (V1), secondary visual cortex (V2), visual area V5/MT and somatosensory cortex (Brodmann areas: BA1, BA2, BA3a, and BA3b), which are available as an automated output of Freesurfer analysis.

### Statistical Analysis

Subject demographics and MwA characteristics were reported using descriptive statistics. Kruskal–Wallis and Mann–Whitney *U*-test were used to compare the data between the groups. The mean cortical thickness of mapped cortical regions was extracted from surface-based morphometry results and exported into the R statistics program. We used GLM and *post-hoc* Tukey tests for comparing the groups in terms of cortical thickness, controlled for the effect of age and sex to avoid spurious results.

## Results

The study included 39 patients with an episodic migraine with typical aura. The characteristics of the patients are reported in Table 2. Overall, 338 MwA attacks were recorded (Table 3) with estimated average aura duration of 47.82 ± 36.1 min [visual aura = 33.72 ± 23.3 (range 10–150); somatosensory aura = 42.62 ± 44.8 (range 10–180); and dysphasic aura = 40.31 ± 41.6 (range 5–180)], headache duration of 7.82 ± 10.7 h (range 1–48) and pain intensity of 6.77 ± 2.1 on the scale from 1 to 10.

**Table 2:** Characteristics of patients.

**Variable**	**Patients (*n* = 39)**
Female, %	29 (74.4%)
Age, mean ± SD (range), in years	38.38 ± 9.8 (24–59)
Age at onset of migraine with aura, mean ± SD (range)	20.49 ± 8.2 (7–38)
Frequency of migraine with aura, mean ± SD (range)	8.67 ± 5.6 (2–28)
Duration of the aura, mean ± SD (range), in minutes	47.82 ± 36.1 (10–180)
Co-occurrence of migraine without aura, %	11 (28.2%)
Familiar history of migraine with aura^a^, %	16 (41.0%)

a *First and second-degree relatives have been considered*.

**Table 3:** Frequency of occurrence of symptoms during migraine with aura attacks.

**Type of symptoms**	**Patients^a^** **(*n* = 39), %**	**Auras** **(*n* = 338), %**
Scotoma	38 (100)	326 (96)
Zig-zag lines	25 (64)	203 (60)
Tunnel vision	8 (21)	42 (12)
Somatosensory aura affecting hand	24 (62)	156 (46)
Somatosensory aura affecting head	21 (54)	141 (42)
Somatosensory aura affecting leg	7 (18)	38 (11)
Visual higher cortical dysfunctions	3 (8)	21 (6)
Somatosensory higher cortical dysfunctions	11 (28)	75 (22)
Dysphasic and/or memory disturbances	18 (46)	103 (30)

a *The described characteristics occurred in the patient at least in one of migraine with aura attacks*.

Seven patients had <6 MwA attacks during the monitored period and therefore they were not included in the group analysis. Comparisons of the groups relative to the patients' characteristics and their MwA features, as well as frequencies of symptoms, are shown in Tables 4, 5, respectively. Scotoma was the most frequently reported symptom in the groups, followed by somatosensory aura in the MwA-C group and zig-zag lines in the MwA-MC and MwA-S groups.

**Table 4:** Comparison of patients' characteristics and their migraine with aura (MwA) features between three groups categorized by MACS.

**Type of symptoms**	**MwA-S** ***n* = 14**	**MwA-MC** ***n* = 9**	**MwA-C** ***n* = 9**	**Statistics**
Female, *n* (%)	9 (64.3)	8 (88.9)	8 (88.9)	*P* = 0.248
Age, mean ± SD (range), in years	36.71 ± 7.7	42.56 ± 9.2	34.22 ± 10.1	*P* = 0.142
Age at onset of migraine with aura, mean ± SD (range)	22.07 ± 7.0	20.67 ± 8.1	16.22 ± 9.9	*P* = 0.256
Frequency of migraine with aura, mean ± SD (range)	9.21 ± 5.7	12.44 ± 7.0	8.11 ± 2.6	*P* = 0.226
Duration of the aura, mean ± SD (range), in minutes	30.00 ± 11.8	59.44 ± 44.9	61.11 ± 49.1	*P* = 0.076
Duration of the headache, mean ± SD (range), in hours	10.57 ± 11.4	7.78 ± 15.2	5.89 ± 7.6	*P* = 0.639
Pain intensity (scale 1–10), mean ± SD	6.21 ± 1.6	6.44 ± 2.1	7.89 ± 2.0	*P* = 0.117
Co-occurrence of migraine without aura	3 (21.4)	4 (44.4)	4 (44.4)	*P* = 0.397
Familiar history of migraine with aura^a^	6 (42.9)	3 (33.3)	4 (44.4)	*P* = 0.872
Scotoma^b^, *n* (%)	14 (100)	9 (100)	8 (88.9)	*P* = 0.267
Zig-zag lines^b^, *n* (%)	11 (78.6)	7 (77.8)	3 (33.3)	*P* = 0.055
Tunnel vision^b^	1 (7.1)	3 (33.3)	3 (33.3)	*P* = 0.206
Somatosensory aura affecting hand^b^, *n* (%)	4 (28.6)	9 (100)	9 (100)	*P* < 0.001 (MwA-S vs. MwA-MC, *p* < 0.001; MwA-S vs. MwA-C, *p* < 0.001)
Somatosensory aura affecting head^b^, *n* (%)	2 (14.3)	7 (77.8)	9 (100)	*P* < 0.001 (MwA-S vs. MwA-MC, *p* = 0.002; MwA-S vs. MwA-C, *p* < 0.001; MwA-MC vs. MwA-C, *p* = 0.031)
Somatosensory aura affecting leg^b^, *n* (%)	1 (7.1)	3 (33.3)	2 (22.2)	*P* = 0.277
Visual higher cortical dysfunctions^b^, *n* (%)	0 (0)	0 (0)	3 (33.3)	*P* = 0.015 (MwA-S vs. MwA-C, *p* < 0.001; MwA-MC vs. MwA-C, *p* < 0.001)
Somatosensory higher cortical dysfunctions^b^, *n* (%)	0 (0)	4 (44.4)	6 (66.7)	*P* = 0.002 (MwA-S vs. MwA-C, *p* = 0.007)
Dysphasic higher cortical dysfunctions^b^, *n* (%)	1 (7.1)	6 (66.7)	9 (100)	*P* < 0.001 (MwA-S vs. MwA-MC, *p* < 0.001; MwA-S vs. MwA-C, *p* < 0.001; MwA-MC vs. MwA-C, *p* < 0.001)
MACS (≥4.5 points), *n* (%)	0 (0)	6 (66.7)	9 (100)	*P* < 0.001 (MwA-S vs. MwA-MC, *p* < 0.001; MwA-S vs. MwA-C, *p* < 0.001; MwA-MC vs. MwA-C, *p* < 0.001)

*MACS, Migraine Aura Complexity Score; MwA-S, patients who have simple auras; MwA-MC, patients who have moderately complex auras; MwA-C, patients who have complex auras*.

a *First and second-degree relatives have been considered*.

b *The described characteristic occurred in the patient at least in one of MwA attacks*.

**Table 5:** Comparison of frequency of MwA symptoms between three groups categorized by the MACS.

**Type of symptoms**	**MwA-S** **(*n =* 129 auras)**	**MwA-MC** **(*n =* 112 auras)**	**MwA-C** **(*n =* 73 auras)**	**Statistics**
Scotoma, *n* (%)	125 (96.9)	112 (100)	73 (100)	*P* = 0.608
Zig-zag lines, *n* (%)	74 (57.4)	94 (83.9)	22 (30.1)	*P* = 0.101
Tunnel vision, *n* (%)	6 (4.7)	18 (16.1)	16 (22.0)	*P* = 0.252
Somatosensory aura affecting hand, *n* (%)	6 (4.6)	79 (70.5)	70 (95.9)	*P* < 0.001 (MwA-S vs. MwA-MC, *p* < 0.001; MwA-S vs. MwA-C, *p* < 0.001)
Somatosensory aura affecting head, *n* (%)	3 (2.3)	64 (57.1)	70 (95.9)	*P* < 0.001 (MwA-S vs. MwA-MC, *p* = 0.002; MwA-S vs. MwA-C, *p* < 0.001)
Somatosensory aura affecting leg, *n* (%)	1 (0.7)	21 (18.7)	16 (22.0)	*P* = 0.250
Visual higher cortical dysfunctions, *n* (%)	0 (0)	0 (0)	21 (28.8)	*P* = 0.017
Somatosensory higher cortical dysfunctions, *n* (%)	0 (0)	23 (20.5)	52 (72.1)	*P* = 0.001 (MwA-S vs. MwA-C, *p* = 0.007)
Dysphasic higher cortical dysfunctions, *n* (%)	2 (1.6)	29 (25.9)	72 (98.6)	*P* < 0.001 (MwA-S vs. MwA-MC, *p* = 0.013; MwA-S vs. MwA-C, *p* < 0.001; MwA-MC vs. MwA-C, *p* = 0.004)
MACS (≥4.5 points), *n* (%)	0 (0)	19 (18.2)	67 (85.0)	*P* < 0.001 (MwA-S vs. MwA-MC, *p* < 0.001; MwA-S vs. MwA-C, *p* < 0.001; MwA-MC vs. MwA-C, *p* < 0.001)

*MACS, Migraine Aura Complexity Score; MwA-S, patients who have migraine with simple auras; MwA-MC, patients who have migraine with moderately complex auras; MwA-C, patients who have migraine with complex auras*.

Comparisons of regions of interest in the visual and somatosensory cortex, as well as cortex involved in a speech, between groups derived from MACS, were shown in Table 6. Patients in the MwA-C and MwA-MC groups have had thicker cortex relative to MwA-S group in the left primary visual cortex (*p* = 0.006; *p* = 0.010), respectively. In addition, patients in the MwA-C group have had thicker cortex relative to MwA-S group in the left secondary visual cortex (*p* = 0.001), right secondary visual cortex (*p* = 0.002), left visual area V5 (*p* = 0.011), right visual area V5 (*p* = 0.013), right somatosensory BA3a cortex (*p* = 0.009), and left somatosensory BA3b cortex (*p* = 0.017).

**Table 6:** Comparison of cortical thickness of the regions of interest in the visual, somatosensory and language cortex between three groups categorized by the MACS.

**Cortical region of interest**	**MwA-S** **(mean ± SD)**	**MwA-MC** **(mean ± SD)**	**MwA-C** **(mean ± SD)**	**Statistics**
Left primary visual cortex	1.397 ± 0.072	1.489 ± 0.066	1.495 ± 0.063	*P* = 0.003 (MwA-S vs. MwA-MC, *p* = 0.010; MwA-S vs. MwA-C, *p* = 0.006)
Right primary visual cortex	1.481 ± 0.106	1.605 ± 0.141	1.590 ± 0.111	*P* = 0.089
Left secondary visual cortex	1.754 ± 0.066	1.828 ± 0.073	1.887 ± 0.095	*P* = 0.005 (MwA-S vs. MwA-C, *p* = 0.001)
Right secondary visual cortex	1.798 ± 0.056	1.889 ± 0.098	1.943 ± 0.115	*P* = 0.002 (MwA-S vs. MwA-C, *p* = 0.002)
Left visual area V5	2.332 ± 0.122	2.450 ± 0.141	2.513 ± 0.152	*P* = 0.008 (MwA-S vs. MwA-C, *p* = 0.011)
Right visual area V5	2.234 ± 0.115	2.331 ± 0.123	2.424 ± 0.201	*P* = 0.010 (MwA-S vs. MwA-C, *p* = 0.013)
Left somatosensory cortex BA1	2.015 ± 0.143	2.040 ± 0.105	2.070 ± 0.207	*P* = 0.477
Right somatosensory cortex BA1	2.104 ± 0.149	2.190 ± 0.159	2.136 ± 0.178	*P* = 0.436
Left somatosensory cortex BA2	2.111 ± 0.107	2.140 ± 0.163	2.217 ± 0.135	*P* = 0.357
Right somatosensory cortex BA2	2.015 ± 0.123	2.104 ± 0.128	2.064 ± 0.126	*P* = 0.209
Left somatosensory cortex BA3a	1.622 ± 0.098	1.649 ± 0.077	1.734 ± 0.115	*P* = 0.081
Right somatosensory cortex BA3a	1.654 ± 0.090	1.704 ± 0.100	1.745 ± 0.082	*P* = 0.040 (MwA-S vs. MwA-C, *p* = 0.009)
Left somatosensory cortex BA3b	1.725 ± 0.116	1.814 ± 0.055	1.859 ± 0.129	*P* = 0.014 (MwA-S vs. MwA-C, *p* = 0.017)
Right somatosensory cortex BA3b	1.583 ± 0.116	1.605 ± 0.106	1.613 ± 0.134	*P* = 0.663
Left BA44	2.558 ± 0.137	2.628 ± 0.105	2.659 ± 0.129	*P* = 0.190
Right BA44	2.502 ± 0.105	2.555 ± 0.076	2.615 ± 0.153	*P* = 0.117
Left BA45	2.409 ± 0.088	2.451 ± 0.165	2.512 ± 0.148	*P* = 0.246
Right BA45	2.440 ± 0.139	2.522 ± 0.132	2.548 ± 0.165	*P* = 0.135

*MACS, Migraine Aura Complexity Score; BA, Brodmann area; MwA-S, patients who have migraine with simple auras; MwA-MC, patients who have migraine with moderately complex auras; MwA-C, patients who have migraine with complex auras*.

Stratification of the patients according to the distinctive manifestations in typical aura yielded 10 patients in MwA-V, 6 patients in MwA-SS and 16 patients in MwA-D group. Comparisons of regions of interest in the visual and somatosensory cortex, as well as cortex involved in a speech, between groups derived according to the distinctive manifestations of typical aura, were shown in Table 7. Patients in the MwA-D and MwA-SS groups have had thicker cortex relative to MwA-V group in the left primary visual cortex (*p* = 0.018; *p* = 0.025), respectively. In addition, patients in the MwA-D group have had thicker cortex relative to MwA-A group in the left secondary visual cortex (*p* = 0.004), right secondary visual cortex (*p* = 0.001), left visual area V5 (*p* = 0.011) and right visual area V5 (*p* = 0.005).

**Table 7:** Comparison of cortical thickness of the regions of interest in the visual, somatosensory and language cortex between three groups categorized by the manifestations of typical aura.

**Cortical region of interest**	**MwA-V** **(mean ± SD)**	**MwA-SS** **(mean ± SD)**	**MwA-D** **(mean ± SD)**	**Statistics**
Left primary visual cortex	1.397 ± 0.077	1.487 ± 0.082	1.470 ± 0.071	*P* = 0.026 (MwA-V vs. MwA-SS, *p* = 0.025; MwA-V vs. MwA-D, *p* = 0.018)
Right primary visual cortex	1.498 ± 0.123	1.614 ± 0.190	1.552 ± 0.099	*P* = 0.056
Left secondary visual cortex	1.751 ± 0.068	1.806 ± 0.076	1.853 ± 0.096	*P* = 0.012 (MwA-V vs. MwA-D, *p* = 0.004)
Right secondary visual cortex	1.783 ± 0.058	1.853 ± 0.093	1.919 ± 0.102	*P* = 0.004 (MwA-V vs. MwA-D, *p* = 0.001)
Left visual area V5	2.313 ± 0.135	2.427 ± 0.072	2.476 ± 0.158	*P* = 0.033 (MwA-V vs. MwA-D, *p* = 0.011)
Right visual area V5	2.200 ± 0.101	2.310 ± 0.109	2.388 ± 0.172	*P* = 0.018 (MwA-V vs. MwA-D, *p* = 0.005)
Left somatosensory cortex BA1	2.018 ± 0.148	1.957 ± 0.133	2.080 ± 0.155	*P* = 0.290
Right somatosensory cortex BA1	2.102 ± 0.167	2.120 ± 0.196	2.166 ± 0.145	*P* = 0.621
Left somatosensory cortex BA2	2.131 ± 0.120	2.073 ± 0.037	2.189 ± 0.157	*P* = 0.478
Right somatosensory cortex BA2	1.988 ± 0.117	2.079 ± 0.126	2.085 ± 0.126	*P* = 0.141
Left somatosensory cortex BA3a	1.637 ± 0.101	1.614 ± 0.085	1.694 ± 0.110	*P* = 0.413
Right somatosensory cortex BA3a	1.643 ± 0.101	1.744 ± 0.096	1.706 ± 0.083	*P* = 0.112
Left somatosensory cortex BA3b	1.728 ± 0.137	1.772 ± 0.066	1.830 ± 0.110	*P* = 0.097
Right somatosensory cortex BA3b	1.578 ± 0.132	1.605 ± 0.123	1.608 ± 0.108	*P* = 0.729
Left BA44	2.541 ± 0.147	2.613 ± 0.082	2.644 ± 0.124	*P* = 0.143
Right BA44	2.488 ± 0.111	2.524 ± 0.081	2.596 ± 0.124	*P* = 0.086
Left BA45	2.394 ± 0.092	2.382 ± 0.040	2.510 ± 0.154	*P* = 0.094
Right BA45	2.423 ± 0.149	2.469 ± 0.092	2.547 ± 0.151	*P* = 0.153

*BA, Brodmann area; MwA-A, patients who have only visual symptoms; MwA-SS, patients who have visual and somatosensory symptoms; MwA-D, patients who have visual, somatosensory and dysphasic symptoms*.

## Discussion

In the present study, we recorded the frequency of MA symptoms in our population of MwA patients and explored a possible difference of patients' characteristics and thickness of cerebral cortex between stratified patients using a Migraine Aura Complexity Score system. The main finding was that patients in the MwA-C and MwA-MC groups had thicker left primary visual cortex relative to the patients from the MwA-S group. In addition, patients in the MwA-C group had thicker cortex in several visual and somatosensory cortical regions with respect to the MwA-S group. Also, stratification into groups using MACS pointed to more cortical regions that should be of interest in further research than stratification into groups according to the distinctive manifestations in the typical aura.

MA usually affects mostly one sensory area of the cerebral cortex producing in the majority of cases only visual symptoms (4, 5). All examined patients in our cohort had visual aura symptoms. MwA can be also manifested as very complex phenomenon including disturbances of multisensory systems in the brain (6) with somatosensory aura symptoms and higher cortical disturbances, which is noted in the MwA-MC and MwA-C groups. Moreover, symptoms during an aura can be very heterogeneous, not just among patients but also in the same patient (5, 18). In our study of 338 MwA attacks that were recorded, the most reported visual symptom was scotoma, followed by zig-zag lines, and tunnel vision, which is also noted in other studies (19). Somatosensory aura was also reported by some patients, mostly affecting the hand and head. Dysphasic symptoms were the most frequent among symptoms of HCDs reported by 46% of patients. These results suggest that patients who have HCDs during the aura are not a small part and such symptoms deserve the attention of physicians who investigates MwA. Recently proposed migraine aura scoring system (14), could help in better categorizing those groups of patients, leading to a phenotype stratification of patients in MwA studies. According to the MACS, patients can be denoted as ones who experience mostly simple auras and those who have mostly complex auras. We believe it is useful to include a third category for those who have a moderately complex aura (i.e., those who suffer from both simple and complex auras). In that way, patients can be stratified more homogeneously in terms of their clinical phenotype.

We used MACS to categorize patients into three groups: MwA-S, MwA-MC, and MwA-C. We, therefore, were able to test our hypothesis that these three groups have different phenotypes and MRI findings. Our opinion is supported by the fact that patients who once experienced complex MA have the potential to experience it again, regardless of whether they have had more frequently reported migraine with a simple aura. Therefore, they should not be labeled as patients whose overall aura is presumed as simple aura, as we implied in our previous study (14). Indeed, in comparison with MwA-C, MwA-MC group reported the similar duration of the aura, the occurrence of the tunnel vision and characteristics of somatosensory aura. However, according to the MACS criteria for complex aura (14), MwA-MC expressed a small percentage of MwA attacks with complex aura. We can only speculate that these results suggest differences in migraineurs brains, which lead patients in MwA-C and MwA-MC groups to express complex manifestation of the aura contrary to patients from MwA-S group. Results also suggest the presence of some inhibitory mechanism in patients from MwA-MC group that prevents the complex manifestation of the MA in most of their attacks, which differentiate them from the MwA-C group.

Furthermore, MACS was used to compare the cortical thickness of regions of interest, which have been linked to MA, in investigated groups. The analysis identified differences in the primary visual cortex, where the cortex was thicker in the MwA-C and MwA-MC groups with respect to the MwA-S. Also, patients in the MwA-C group had thicker cortex relative to MwA-S group in several visual and secondary somatosensory cortical regions, suggesting that MA could be associated with different ways of aberrant brain functional organization (20). These differences were not observed between the MwA-C and MwA-MC groups, which could explain the overlap of the clinical characteristics of these two groups. However, this finding should be further investigated with a larger cohort of patients and with multimodal neuroimaging techniques to strengthen the interpretation of changes in the migraineurs brain. Moreover, previous neuroimaging studies of MwA implicate different brain regions as biomarkers for MwA (13, 21–23), which could be explained by the lack of adequate stratification of patients in which MACS may help. Also, these heterogeneous neuroimaging findings could represent specific brain networks for subtypes of MA (24). This can be further strengthened by the finding that stratification of patients according to the distinctive manifestations in typical aura pointed to the same results, which challenges the point of view that patients who have only visual symptoms and someone who has visual and somatosensory or dysphasic aura should be equally weighted and placed in the same group. Moreover, stratifications using MACS point to more cortical regions, allocated to the somatosensory cortex, that could be involved in a different experience of aura in MwA patients. Of course, strong similarities between the investigated two modalities of stratification of MwA patients and an additional contribution of MACS model in neuroimaging studies should be confirmed by other independent investigators. Anyhow, the neuroimaging findings from our study suggest that MACS can be successfully used for the stratification of patients in studies investigating the difference in cortical thickness among distinct phenotypes of MwA patients. Moreover, the fact that selected patients regularly completed questionnaire after each MwA attack and no one withdrew from the study, suggest that the MACS can be properly implemented and fulfill its intended use.

Altogether, if we would try to describe phenotypes of patients from those three groups, the results suggest that the patients from the MwA-S group have a shorter duration of the whole aura, longer duration of the headache, rare occurrence of the tunnel vision, somatosensory aura and symptoms of HCDs, as well as thinner cerebral cortex in general, with respect to the MwA-MC and MwA-C groups. Shorter duration of the aura in patients who did not experience HCDs was previously noticed (6). This finding supports the idea that the duration of the aura may depend on the site in which the CSD originates (9) and the adaptive capacity of affected regions which can abort CSD through other cortical regions, thus avoiding symptoms of somatosensory aura and HCDs. On the other hand, patients from the MwA-C group started to experience MwA at a younger age, have less frequent MwA attacks although more severe headaches, rare occurrence of the zig-zag lines in their visual field but more frequent occurrence of the HCDs, as well as thicker cerebral cortex in general, when compared to the MwA-MC and MwA-S groups. This demonstrates that cerebral cortex is a hallmark for the investigation of the pathophysiology of a complex MA and require further sub-phenotypes investigation in order to link HCDs and changes in cortical thickness. Patients from the MwA-MC group are prone to more frequent MwA attacks and more common occurrence of the zig-zag lines with respect to the MwA-C and MwA-S groups. Also, they have more similarity to the MwA-C group in terms of duration of the aura and occurrence of the tunnel vision. Anyhow, studies including a higher number of patients per group should provide a more detailed profile of such identified phenotypes.

A limitation of the study is that the sample size of three subgroups is relatively small for definitive conclusions. However, the strength of the study is that participants were carefully divided into homogenous groups according to their clinical phenotypes and that neuroimaging results were strongly comparable with findings from investigation of groups stratified according to the distinctive manifestations in the typical aura. Moreover, our patients did not present any comorbidity and did not use migraine preventive therapy that could have influenced the investigation. Also, we based our discussion on the results that were not corrected for multiple comparisons. Although this could lead to false-positive findings, correction for multiple comparisons increases the risk of generating false-negative findings (25), which could underestimate subtle differences in the investigated groups. Because of that, we think that is important to show and discuss uncorrected data for multiple comparisons to achieve better methodological solutions which will allow using a better strategy for investigation of MA pathophysiology and new targets for treating it. Finally, the results of this study should be confirmed using a new and independent cohort of subjects.

Our results show that the newly developed MACS can be used for the stratification of MwA patients and identifying their phenotypes, herewith allowing the better investigation of changes in migraineurs' brains. Further efforts toward a better system for stratification of MwA patients are needed to provide new knowledge about complex pathological mechanisms of MA and their influence on the brain plasticity. Thus, the MACS may help in revealing new therapeutic targets and evaluation of the efficiency of MA treatment.

## Data Availability Statement

The datasets generated for this study are available on request to the corresponding author.

## Ethics Statement

This study was approved by the Medical Ethics Committee of the Neurology Clinic, Clinical Center of Serbia, and was conducted in accordance with the Declaration of Helsinki. Informed consent forms were completed by all the participants after receiving an explanation of the study.

## Author Contributions

IP contributed to the study aim, design, acquisition, analysis, interpretation, and drafting of the manuscript. MV contributed to the interpretation and drafting of the manuscript. MD contributed to acquisition and analysis. JZ-T contributed to interpretation and critically revised manuscript. All authors read and approved the final manuscript.

### Conflict of Interest

The authors declare that the research was conducted in the absence of any commercial or financial relationships that could be construed as a potential conflict of interest.

## Abbreviations

CSD: Cortical spreading depression
GLM: General linear model
MA: migraine aura
MACS: Migraine Aura Complexity Score
MRI: magnetic resonance imaging
MwA: migraine with aura
MwA-C groups: patients who have a migraine with complex aura
MwA-MC group: patients who have a migraine with moderately complex aura
MwA-S group: patients who have a migraine with simple aura
MwA-A group: patients who have only visual symptoms
MwA-SS group: patients who have visual and somatosensory symptoms
MwA-D group: patients who have visual, somatosensory, and dysphasic symptoms.

## References

[1] GBD 2016 Headache Collaborators Global, regional, and national burden of migraine and tension-type headache, 1990–2016: a systematic analysis for the Global Burden of Disease Study 2016. Lancet Neurol. (2018) 17:954–76. 10.1016/S1474-4422(18)30322-3 30353868 PMC6191530

[2] Steiner TJ Stovner LJ Vos T Jensen R Katsarava Z. Migraine is first cause of disability in under 50s: will health politicians now take notice? J Headache Pain. (2018) 19:17. 10.1186/s10194-018-0846-2 29468450 PMC5821623

[3] Rasmussen BK Olesen J. Migraine with aura and migraine without aura: an epidemiological study. Cephalalgia. (1992) 12:221–8. 10.1046/j.1468-2982.1992.1204221.x 1525797

[4] Headache Classification Committee of the International Headache Society (IHS) The international classification of headache disorders, 3rd edition. Cephalalgia. (2018) 38:1–211. 10.1177/0333102417738202 29368949

[5] Viana M Sances G Linde M Ghiotto N Guaschino E Allena M. Clinical features of migraine aura: results from a prospective diary-aided study. Cephalalgia. (2017) 37:979–89. 10.1177/0333102416657147 27573009

[6] Petrusic I Zidverc-Trajkovic J Podgorac A Sternic N. Underestimated phenomena: higher cortical dysfunctions during migraine aura. Cephalalgia. (2013) 33:861–7. 10.1177/0333102413476373 23430982

[7] Swanson SA Zeng Y Weeks M Colman I. The contribution of stress to the comorbidity of migraine and major depression: results from a prospective cohort study. BMJ Open. (2013) 3:e002057. 10.1136/bmjopen-2012-002057 23474788 PMC3612807

[8] Hadjikhani N Sanchez del Rio M Wu O Schwartz D Bakker D Fischl B. Mechanisms of migraine aura revealed by functional MRI in human visual cortex. Proc Natl Acad Sci USA. (2001) 98:4687–92. 10.1073/pnas.071582498 11287655 PMC31895

[9] Petrusic I Zidverc-Trajkovic J. Cortical spreading depression: origins and paths as inferred from the sequence of events during migraine aura. Funct Neurol. (2014) 29:207–12. 10.11138/FNeur/2014.29.3.207 25473742 PMC4264789

[10] Chong CD Schwedt TJ Dodick DW. Migraine: what Imaging Reveals. Curr Neurol Neurosci Rep. (2016) 16:64. 10.1007/s11910-016-0662-5 27181270

[11] Yang H Zhang J Liu Q Wang Y. Multimodal MRI-based classification of migraine: using deep learning convolutional neural network. Biomed Eng Online. (2018) 17:138. 10.1186/s12938-018-0587-0 30314437 PMC6186044

[12] Szabó N Faragó P Király A Veréb D Csete G Tóth E. Evidence for plastic processes in migraine with aura: a diffusion weighted MRI study. Front Neuroanat. (2018) 11:138. 10.3389/fnana.2017.00138 29387002 PMC5776127

[13] Petrusic I Dakovic M Kacar K Zidverc-Trajkovic J. Migraine with aura: surface-based analysis of the cerebral cortex with magnetic resonance imaging. Korean J Radiol. (2018) 19:e86. 10.3348/kjr.2018.19.4.767 29962883 PMC6005951

[14] Petrusic I Viana M Dakovic M Goadsby PJ Zidverc-Trajkovic J. Proposal for a migraine aura complexity score. Cephalalgia. (2018) 39:732–41. 10.1177/0333102418815487 30458631

[15] Petrušić I Daković M Kačar K Mićić O Zidverc-Trajković J. Migraine with aura and white matter tract changes. Acta Neurol Belg. (2018) 118:485–91. 10.1007/s13760-018-0984-y 30006859

[16] Dale AM Fischl B Sereno MI. Cortical surface based analysis I: segmentation and surface reconstruction. Neuroimage. (1999) 9:179–94. 10.1006/nimg.1998.0395 9931268

[17] Fischl B. FreeSurfer. Neuroimage. (2012) 62:774–81. 10.1016/j.neuroimage.2012.01.021 22248573 PMC3685476

[18] Hansen JM Goadsby PJ Charles AC. Variability of clinical features in attacks of migraine with aura. Cephalalgia. (2016) 36:216–24. 10.1177/0333102415584601 25944814

[19] Queiroz LP Friedman DI Rapoport AM Purdy RA. Characteristics of migraine visual aura in Southern Brazil and Northern USA. Cephalalgia. (2011) 31:1652–8. 10.1177/0333102411430263 22116942

[20] Schwedt TJ Chong CD. Functional imaging and migraine: new connections? Curr Opin Neurol. (2015) 28:265–70. 10.1097/WCO.0000000000000194 25887764 PMC4414904

[21] DaSilva AF Granziera C Snyder J Hadjikhani N. Thickening in the somatosensory cortex of patients with migraine. Neurology. (2007) 69:1990–5. 10.1212/01.wnl.0000291618.32247.2d 18025393 PMC3757544

[22] Messina R Rocca MA Colombo B Valsasina P Horsfield MA Copetti M. Cortical abnormalities in patients with migraine: a surface-based analysis. Radiology. (2013) 268:170–80. 10.1148/radiol.13122004 23533286

[23] Gaist D Hougaard A Garde E Reislev NL Wiwie R Iversen P. Migraine with visual aura associated with thicker visual cortex. Brain. (2018) 141:776–85. 10.1093/brain/awx382 29360944

[24] Chong CD Gaw N Fu Y Li J Wu T Schwedt TJ. Migraine classification using magnetic resonance imaging resting-state functional connectivity data. Cephalalgia. (2017) 37:828–44. 10.1177/0333102416652091 27306407

[25] Sangseok L Dong KL What is the proper way to apply the multiple comparison test? Korean J Anesthesiol. (2018) 71:353–60. 10.4097/kja.d.18.00242 30157585 PMC6193594

